# Scimitar Syndrome in Adolescence

**DOI:** 10.1002/rcr2.70815

**Published:** 2026-11-02

**Authors:** Shan Kai Ing, Yih Hoong Lee, Joyce Mei Juin Yip, Jennie Geok Lim Tan, Nga Hung Ngu

**Affiliations:** ^1^ Respiratory Unit, Department of Medicine Hospital Sibu Sibu Sarawak Malaysia; ^2^ Hospital Kanowit Kanowit Sarawak Malaysia; ^3^ Department of Radiology Hospital Sibu Sibu Sarawak Malaysia

**Keywords:** adolescent, computed tomography, partial anomalous pulmonary venous return, right lung hypoplasia, scimitar syndrome

## Abstract

Scimitar syndrome has infantile and childhood/adult variants. We describe an adolescent girl with recurrent wheeze in whom chest radiography and multimodality CT demonstrated the characteristic scimitar vein and associated bronchovascular abnormalities, with normal right ventricular function and no pulmonary hypertension.

## Graphical abstract

Scimitar syndrome may present beyond infancy with non‐specific respiratory symptoms. Chest radiography and CT demonstrated right lung hypoplasia, cardiac dextroposition, associated bronchovascular anomalies and anomalous pulmonary venous drainage into the inferior vena cava, establishing the diagnosis.

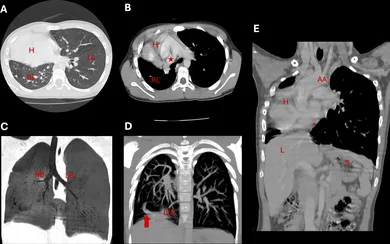

A 14‐year‐old girl with allergic rhinitis and bronchial asthma was referred from a district hospital for evaluation of a 2‐week history of dry cough, wheeze and dyspnoea. Examination showed tachycardia, generalised rhonchi and reduced air entry over the right lung field; oxygen saturation was 96% on room air.

Chest radiography demonstrated reduced right‐lung volume, ipsilateral tracheomediastinal shift, cardiac dextroposition and a tubular right lower‐zone opacity (Figure 1). Contrast‐enhanced CT showed right lung hypoplasia with absent right upper lobe, compensatory left‐lung hyperinflation, hypoplastic right pulmonary artery, a hyparterial bilobed right bronchus and anomalous pulmonary venous drainage into the diaphragmatic inferior vena cava, producing the classic scimitar sign (Figure 2). These findings were diagnostic of the childhood/adult variant of scimitar syndrome, which is generally associated with lower morbidity than the infantile form [1, 2].

**FIGURE 1:**
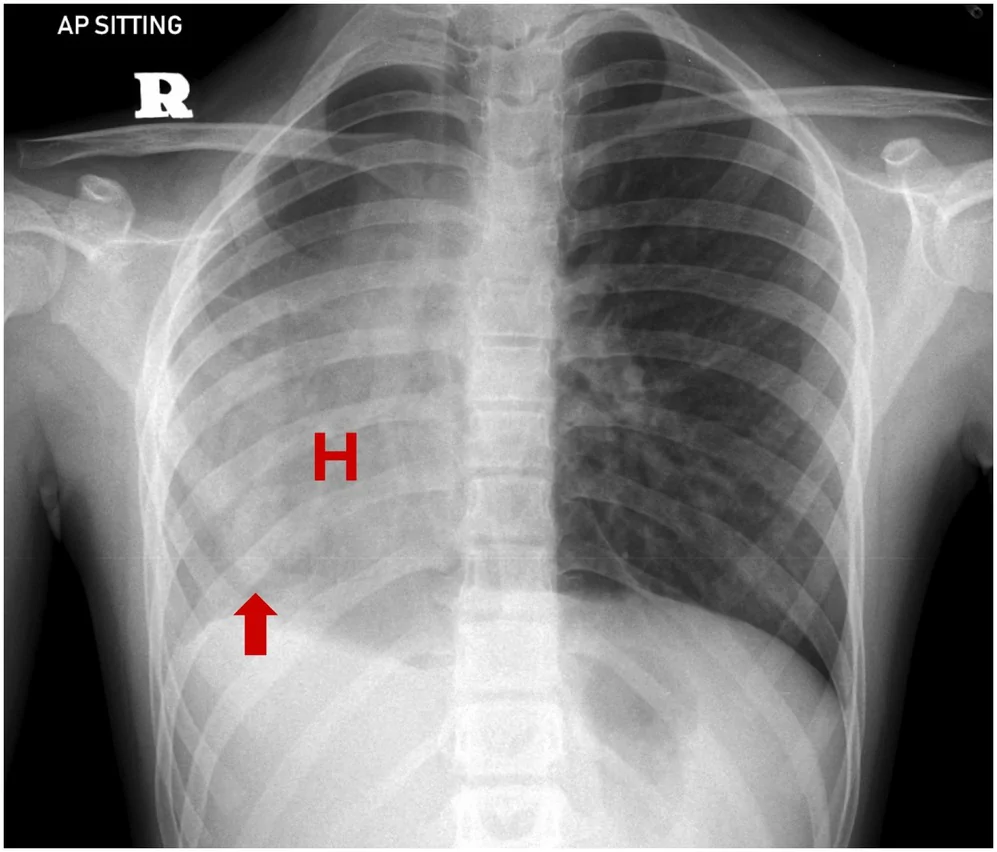
Chest radiograph demonstrating reduced right‐lung volume, ipsilateral tracheomediastinal shift, cardiac dextroposition (H) and a tubular right lower‐zone opacity (arrow).

**FIGURE 2:**
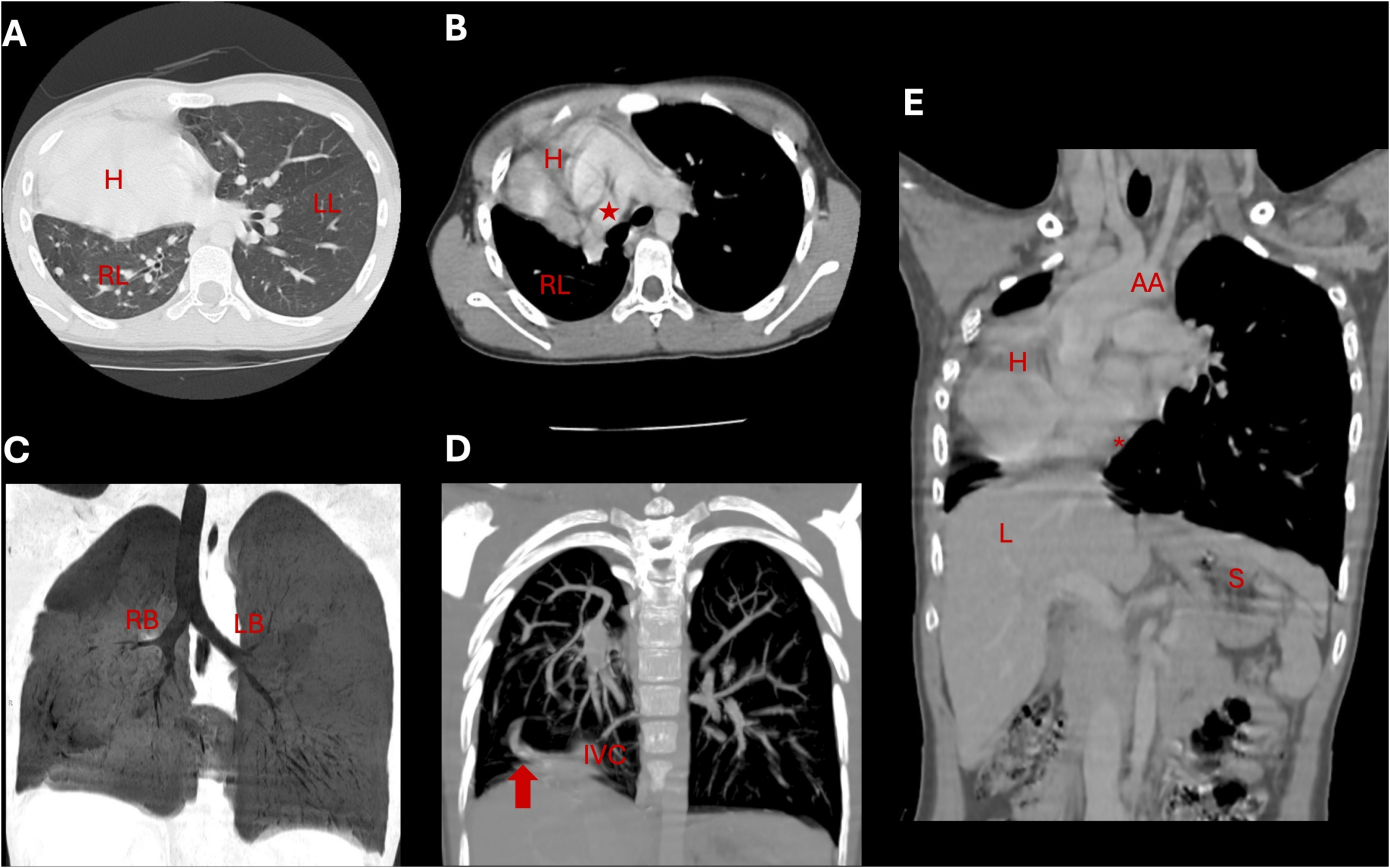
Composite CT images demonstrating: (A) right lung (RL) hypoplasia with absence of the right upper lobe, compensatory hyperinflation of the left lung (LL) and cardiac dextroposition (H); (B) hypoplastic right pulmonary artery (star); (C) coronal minimum‐intensity‐projection image showing a hyparterial bilobed right bronchus (RB) with branching pattern similar to the left bronchus (LB); and (D) coronal maximum‐intensity‐projection image showing anomalous pulmonary venous drainage into the diaphragmatic inferior vena cava (arrow), producing the classic scimitar sign; (E) coronal CT showing left‐sided cardiac apex (asterisk) and normal left‐sided aortic arch (AA) in keeping with dextroposition of the heart (H). The liver (L) is right‐sided and the stomach (S) left‐sided, consistent with situs solitus.

Spirometry was normal. Echocardiography showed normal right ventricular size and function, normal chamber dimensions and no evidence of pulmonary hypertension. The acute presentation was primarily attributable to asthma and resolved completely with asthma treatment. She was referred for respiratory and cardiology follow‐up.

## Author Contributions

Shan Kai Ing conceived the report, collected and interpreted the clinical information, drafted the manuscript and serves as guarantor. Yih Hoong Lee contributed to the patient's clinical assessment and management and critically revised the manuscript. Joyce Mei Juin Yip referred the patient, provided the initial clinical information and contributed to manuscript revision. Jennie Geok Lim Tan interpreted the radiological findings, selected and prepared the images, and critically revised the manuscript. Nga Hung Ngu contributed to the patient's respiratory management and critically revised the manuscript for important intellectual content. All authors reviewed and approved the final manuscript and agree to be accountable for all aspects of the work.

## Funding

The authors have nothing to report.

## Ethics Statement

Ethics committee approval was not required for this single‐patient clinical image report. The manuscript was prepared in accordance with recognised ethical standards for publication of clinical material.

## Consent

The authors declare that written informed consent was obtained from the patient's parent or legal guardian for publication of this manuscript and accompanying images using the consent form provided by the Journal.

## Conflicts of Interest

The authors declare no conflicts of interest.

## Data Availability

Data sharing not applicable to this article as no datasets were generated or analysed during the current study.
